# Epidemiology, management and outcome of acute respiratory distress syndrome in Sub-Saharan Africa: a systematic review

**DOI:** 10.1177/20542704251390024

**Published:** 2025-11-06

**Authors:** Valentina Camarda, Robert F. Miller

**Affiliations:** 1 Institute for Global Health, 204274 University College London, London WC1E 6JB, UK

**Keywords:** intensive care, ARDS, respiratory failure, mechanical ventilation, sub-Saharan Africa

## Abstract

**Objectives:**

To evaluate the incidence, management, and outcomes of Acute Respiratory Distress Syndrome (ARDS) in Sub-Saharan Africa (SSA), and to identify challenges related to healthcare infrastructure and resource availability.

**Design:**

Systematic review of published studies on ARDS in SSA.

**Setting:**

Studies conducted across hospitals and intensive care units in 11 countries within Sub-Saharan Africa between 2000 and 2024.

**Participants:**

Adult patients diagnosed with ARDS.

**Main Outcome Measures:**

Prevalence of ARDS, patient demographics, management strategies, availability of critical care resources, and mortality rates.

**Results:**

Thirteen studies met the inclusion criteria. ARDS prevalence varied widely, ranging from 2.4% to 100%. The Kigali modification of the Berlin criteria was most frequently applied, reflecting limited access to chest radiography and arterial blood gas analysis. Pneumonia, sepsis, and trauma were the predominant causes, with infectious diseases such as HIV, tuberculosis, and malaria contributing substantially. Access to invasive mechanical ventilation and other critical care resources was limited. Reported mortality rates ranged from 22% to 77%.

**Conclusions:**

ARDS represents a major but under-recognised cause of morbidity and mortality in SSA. Resource limitations, including inadequate diagnostic capacity and restricted access to mechanical ventilation, likely contribute to poor outcomes. Efforts to strengthen critical care infrastructure, provide targeted training, and adapt diagnostic criteria for low-resource environments are urgently needed. Further research should explore regional variations and context-appropriate interventions to improve ARDS care across SSA.

## Background

Acute respiratory distress syndrome (ARDS) is a life-threatening condition characterised by increased permeability of the alveolo-capillary membrane, leading to lung oedema, decreased lung compliance due to non-aerated lung tissue, and increased dead space, which results in hypoxaemia and hypercapnia.^1,2^ Globally, ARDS accounts for approximately 10% of all intensive care unit (ICU) admissions and 23% of patients requiring invasive mechanical ventilation (IMV), with mortality rates for severe ARDS being as high as 45%.^3^

Despite the likely common occurrence of ARDS in resource-limited settings, data on its prevalence and outcomes are scarce. This data gap is a common issue in critical care research in low-resource environments.^4^ Moreover, the Berlin Definition of ARDS requires diagnostic tools and treatment capabilities that are often unavailable in these settings, limiting the recognition and quantification of ARDS.^5,6^ To address these constraints, the Kigali modification of the Berlin Definition was proposed for use in low-resource settings.^5^ This removes the need for positive end-expiratory pressure (PEEP) and defines ARDS based on the presence of bilateral opacities seen on lung ultrasound (LUS) or chest X-ray (CXR), and hypoxia. Oxygen saturation measured by pulse oximetry (SpO_2_) is used instead of arterial blood gases (ABGs), with a threshold SpO_2_/FiO_2_ (fraction of inspired oxygen) ratio of ≤315. In 2021, a global consensus incorporated the Kigali definition into a new global definition of ARDS.^7^

Sub-Saharan Africa (SSA), a region encompassing 48 countries, faces unique healthcare challenges that may influence the epidemiology and outcomes of ARDS. High rates of infection and trauma, which are common ARDS risk factors, coexist with widespread poverty, malnutrition, and limited healthcare access.^4,8^

Despite the high burden of potential risk factors, data on the epidemiology, management, and outcomes of ARDS in SSA remain scarce. This systematic review aims to address this data gap.

## Methods

### Search strategy

A comprehensive literature search was conducted to identify studies related to the epidemiology, management and outcome of ARDS in SSA. The databases searched included PubMed, Cochrane Library, and Embase. The search was performed in July 2024 and was limited to studies published in English. The search strategy involved a combination of keywords and MeSH (Medical Subject Headings) terms, including ‘ARDS’, ‘Acute Respiratory Distress Syndrome’, ‘Sub-Saharan Africa’, ‘epidemiology’, ‘management’ and ‘outcomes’. In addition, specific SSA country names were included to capture region-specific data.

Filters were applied to include only studies involving adult populations from 2000 to 2024. This time frame was chosen to ensure that the included studies reflect contemporary medical practices, diagnostic criteria, and treatment standards. To adapt to the evolving definitions of ARDS, the search strategy also included alternative terms used in earlier literature, such as ‘acute lung injury’, ‘ALI’ and ‘respiratory distress syndrome’.^9^ The detailed search strategy is shown in the supplemental material.

### Study selection process

The study selection process followed the PRISMA guidelines. Studies were included if they met the following criteria: (1) focused on the epidemiology, management, or outcomes of ARDS in the context of SSA; (2) were original research articles, systematic reviews, or meta-analyses; (3) involved adult populations; and (4) were published in English. Exclusion criteria were: (1) studies conducted outside of SSA; (2) case reports, editorials, opinion pieces, and conference abstracts; and (3) articles not available in English.

All titles and abstracts identified were screened independently by two reviewers against the eligibility criteria. Any disagreements were resolved through discussion until consensus was reached.

### Data extraction

A standardised data extraction form was developed to collect relevant information from the selected studies, including author details, publication year, study design, sample size, population characteristics, oxygen supplementation type, use of supportive therapies, key findings on the epidemiology, management, and outcomes of ARDS, and conclusions. Each publication was also evaluated for its strengths and limitations. For cross-sectional studies, the Joanna Briggs Institute (JBI) critical appraisal tool was used to assess methodological quality, while the Newcastle-Ottawa Scale (NOS) was applied for cohort studies (see Supplementary file). Data extraction was performed by the first author and independently verified by the supervising author.

### Data synthesis

The presentation of data in this review was systematically organised to enhance clarity. Data from the selected studies were categorised into key themes, including epidemiological characteristics, clinical management, and patient outcomes. To aid in the interpretation of findings, tables were utilised to summarise essential metrics, such as prevalence rates, demographics, and treatment strategies.

## Results

### Study selection

**Figure 1.**
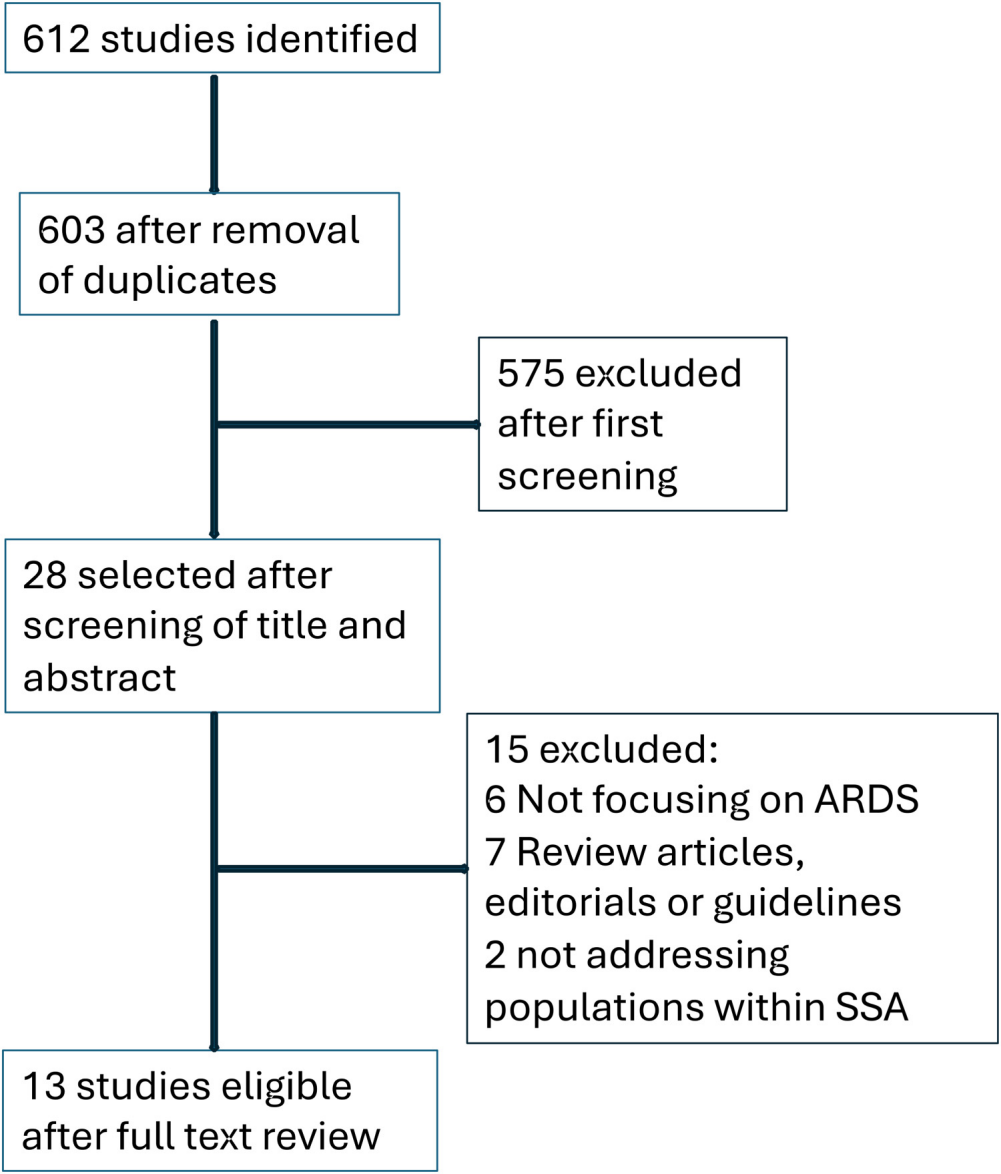
Study selection.

The initial search identified 612 studies (PubMed: 378, Cochrane Library: 63, Embase: 180). After removing duplicates, 603 unique articles were screened by title and abstract, yielding 28 studies for full-text review. The most common reasons for exclusion were lack of focus on ARDS, being review articles or editorials, or not addressing populations within SSA. Following the full-text review, 13 studies were deemed eligible for inclusion in the final analysis (Figure 1).

### Study characteristics

The 13 studies selected span from 2000 to 2024 and focus on the prevalence, risk factors, management, and outcomes of ARDS in SSA. Most studies were cross-sectional or cohort studies, focusing on ICU admissions, respiratory support strategies, and mortality. A systematic review and meta-analysis evaluating ICU mortality and determinants was also included.

The studies were conducted in 11 countries: Rwanda, Zambia, Ghana, Ethiopia, Gabon, Sierra Leone, Tanzania, Malawi, South Africa, Uganda and Guinea. Patient populations ranged from as few as 19 patients to over 7000. Most included adults (≥18 years), though some extended to younger populations. Patient groups varied widely, including mechanically ventilated patients, COVID-19 and H1N1 influenza cases, obstetric patients, and general ICU populations. Table 1 summarises the characteristics of each study.

**Table 1.** Characteristics of studies included in the systematic review.

**Study**	**Country**	**Sample** s**ize**	**Population**	**Design**	**Primary outcome**
**Non-COVID-19 studies**					
**Koegelenberg *et al.* (2010)**	South Africa	19	H1N1-related ARDS patients	Cross-sectional study	Epidemiological characteristics and outcomes of H1N1-related ARDS patients
**Riviello *et al.* (2015)**	Rwanda	1046	Hospitalised patients	Prospective cohort study	ARDS prevalence and outcomes
**Osei-Ampofo *et al.* (2018)**	Ghana	102	Mechanically ventilated patients	Prospective observational study	Incidence of ARDS requiring IMV in emergency centres and ICU
**Kwizera *et al.* (2020)**	Uganda	7300	Acute hypoxemic respiratory failure patients	Prevalence study	Prevalence of AHRF in critically ill patients
**Pisani *et al.* (2020)**	Sierra Leone	166	Critically ill parturients	Prospective cohort study	Proportion of parturients with pulmonary complications detectable by LUS
**Freercks *et al.* (2022)**	South Africa	712	Medical ICU patients	Retrospective cohort study	ICU admissions and outcomes
**Kwizera *et al.* (2022)**	Uganda	231	Mechanically ventilated patients	Prevalence study	ARDS prevalence in mechanically ventilated patients
**Endeshaw *et al.* (2022)**	Ethiopia	8915	ICU patients	Systematic review and meta-analysis	Prevalence of ICU mortality and determinants
**Chang *et al.* (2023)**	Multinational (SSA)	7385	Multiple cohorts	Retrospective multicohort study	Characteristics and outcomes of SRD in hospitalised patients
**COVID-19 studies**					
**Donamou *et al.* (2021)**	Guinea	140	Critically ill patients with COVID-19	Cross-sectional study	Identification of COVID-related mortality factors in ICU
**Tolossa *et al.* (2022)**	Ethiopia	318	COVID-19 patients	Cross-sectional study	Prevalence and risk factors for ARDS in COVID-19 patients
**Arnold-Day *et al.* (2022)**	South Africa	380	COVID-19 patients with ARDS requiring IMV	Prospective cohort study	Characteristics and outcomes of COVID-19 patients
**Kwizera *et al.* (2023)**	Uganda	499	COVID-19 patients with ARDS	Prospective cohort study	Respiratory support and outcomes of COVID-19 patients with ARDS

ARDS: acute respiratory distress syndrome; IMV: invasive mechanical ventilation; ICU: intensive care unit; AHRF: acute hypoxic respiratory failure; LUS: lung ultrasound; SSA: Sub-Saharan Africa; SRD: severe respiratory distress.

Statistical methods ranged from basic descriptive analyses to multivariable regression models assessing factors associated with poor outcomes. Common challenges included missing data, selection bias, and diagnostic difficulties in resource-limited settings. Study quality, assessed using NOS and JBI critical appraisal frameworks, was generally high, with most employing validated diagnostic criteria such as LUS. The NOS score of the cohort studies was between 7 and 9, while the JBI score was between 6 and 8. In the systematic review, only studies with an NOS score of 5 or higher were included (see Supplementary file).

## Synthesis of results

### Patient demographics and risk factors

The median age of patients with ARDS ranged from 37 to 54 years. Male patients were disproportionately affected, accounting for between 51% and 72% of ARDS cases in most cohorts. However, exceptions were observed in some settings. For instance, in a study of ARDS due to H1N1 influenza in South Africa, females were the majority (78.9%).^10^ Similarly, in another study from South Africa, 50.5% of COVID-19 patients with ARDS were female.^11^

Co-morbidities such as diabetes mellitus (DM) and hypertension (HTN) feature prominently in many studies, with DM being present in up to 47.6% of patients in some reports and HTN reported in 48.2% of patients.^11,12^ Other notable co-morbidities include human immunodeficiency virus (HIV), with a prevalence across studies ranging from 8.6% to 46%, chronic heart failure, chronic obstructive pulmonary disease (COPD), and a history of tuberculosis (TB).

Predisposing factors for ARDS ranged widely, with infectious causes such as pneumonia and sepsis being major contributors across the studies. Notably, sepsis was reported in 22.1%–30.4%, and pneumonia was a leading cause in several settings, with rates up to 80%.^13,14^ Trauma and postoperative complications were also common predisposing factors.

Pregnancy was a particularly notable risk factor, especially in the context of H1N1- and COVID-19-related ARDS, where pregnant women were at significantly higher risk.^10,15^ Table 2 summarises patient demographics and risk factors for ARDS.

**Table 2.** Diagnostic criteria and prevalence of ARDS, patient demographics and predisposing factors for ARDS.

**Study**	**Diagnostic criteria used**	**Prevalence/incidence of ARDS (%)**	**ARDS age (years)**	**ARDS male (%)**	**Main co-morbidities**	**ARDS predisposing factors**
**Non-COVID-19 studies**						
**Koegelenberg *et al.* (2010)**	1994 AECC definition of ARDS	All ARDS patients	Mean 39.5 (SD 14.8)	21.1%	DM (31.58%) Obesity (21.05%) Immunosuppression (15.79%) HIV (15.79%) COPD (5.26%) Active TB (10.53%) Previous TB (10.53%)	H1N1, pregnancy (31.58%)
**Riviello *et al.* (2015)**	Kigali	4% Incidence	Median 37 (IQR 26–49)	54.4%	Not reported	Infection (44%), trauma (29.4%), surgery (25%), stroke (4.4%), other (13.2%)
**Osei-Ampofo *et al.* (2018)**	Kigali	2.4% Incidence	Not reported	Not reported	Not reported	Pneumonia, surgery
**Kwizera *et al.* (2020)**	Kigali	4.4% Prevalence	Median 38 (IQR 27–52)	60%	Use of firewood (15%), smoking (15%), asthma (1.8%), previous acute lung injury (5.5%), HIV (30.8%)	Pneumonia (80%), cardiac disease (8.5%), neurological disease (25.9%), sepsis (30.4%)
**Pisani *et al.* (2020)**	Kigali	3.6% Prevalence	Not reported	0	Not reported	Obstetrics, sepsis
**Kwizera *et al.* (2022)**	Kigali	70.56% Prevalence	Mean 45.1 (SD 18.3)	60%	DM (3.68%) Cancer (4.91%) HIV (11.66%) Malaria (2.45%)	TBI (20.25%) Sepsis (22.09%) Pneumonia (3.68%) Postoperative (17.18%) Neurological disease (7.36%) Obstetric (7.98%) Cardiac disease (4.30%)
**Freercks *et al.* (2022)**	Berlin criteria	4.6% Prevalence	Not reported	Not reported	Not reported	Not reported
**Chang *et al.* (2023)**	WHO criteria SRD	12.8%	median 37 (IQR 27–53)	51%	HIV (46%)	Infective (60%), undifferentiated cause (40%)
**COVID-19 studies**						
**Tolossa *et al.* (2022)**	Kigali	32% Prevalence	<65 83%	72.5%	HTN (48.23%), DM (29.08%), chronic heart failure (15.60%), stroke (0.71%), TB (9.93%), HIV (10.64%), asthma (12.06%), CKD (2.84%), others (7.09%)	COVID-19
**Arnold-Day *et al.* (2022)**	Berlin criteria	All ARDS patients	Median 51 (IQR 17–71)	49.5%	None (18.3%), HTN (46.3%), DM (47.6%), HIV (10.5%)	COVID-19
**Kwizera *et al.* (2023)**	Kigali	All ARDS patients	Median 54 (IQR 42–65)	68.1%	HTN (30.9%), DM (18.4%), HIV (8.6%), other 29 (5.8%)	COVID-19, pregnancy (2.2%)

ARDS: acute respiratory distress syndrome; SD: standard deviation; DM: diabetes mellitus; HIV: human immunodeficiency virus; COPD: chronic obstructive pulmonary disease; TB: tuberculosis; IQR: interquartile range; WHO: World Health Organization; SRD: severe respiratory distress; HTN: hypertension; CKD: chronic kidney disease; TBI: traumatic brain injury; AECC: American-European Consensus Conference.

### Diagnostic criteria and prevalence of ARDS in SSA

Diagnostic criteria varied across studies. Many adopted the Kigali modification of the Berlin definition, using pulse oximetry and LUS for diagnosis due to limited access to ABG sampling. Older studies,^10^ used the 1994 American-European Consensus Conference definition of acute lung injury (ALI), while some relied on the WHO (World Health Organization)–SRD (severe respiratory distress) syndrome criteria.^16^ One study defined acute hypoxic respiratory failure (AHRF) based on clinical signs and pulse oximetry.^14^

ARDS prevalence ranged from 2.4% to 100% (Table 2). The highest prevalence, 70.6%, was observed in Uganda among mechanically ventilated patients,^13^ while emergency department cases had a prevalence of 4.5%.^15^ COVID-19-related ARDS prevalence reached 32% in Ethiopia.^12^ In South Africa, 4.6% of ICU admissions were with ARDS.^17^ In Sierra Leone, in critically ill parturient patients, the prevalence of ARDS was 3.6%,^18^ while a multicohort study estimated WHO-SRD prevalence at 12.8%.^16^

### Oxygen support and adjunctive strategies

Oxygen support varied, ranging from face masks and high-flow nasal oxygen (HFNO) to continuous positive airway pressure (CPAP), non-invasive ventilation (NIV), and IMV. IMV availability was often limited and inconsistently reported (see Supplementary file). In Uganda, only 6% of ARDS patients received IMV.^14^ Similarly, in Ethiopia, 73.4% of severe COVID-19 patients received intranasal oxygen, while IMV was not reported.^12^ In contrast, in Rwanda, 30.9% of patients received IMV.^5^

For COVID-19-related ARDS in a study from Uganda, 35.5% received conventional oxygen, 9.4% HFNO, 4.8% CPAP, 14.4% NIV, and 36.1% received IMV.^15^ Some studies focused solely on patients receiving IMV, while others lacked description of oxygen support, highlighting inconsistencies in reporting (see Supplementary file).^11,15,19^

Ventilation strategies were detailed in three studies.^11,15,19^ Tidal volumes of 6.9–8.7 mL/kg of ideal body weight were reported^19^; however, proning (three studies^11,15,19^), neuromuscular blockade (two studies^11,19^) and veno-venous extracorporeal membrane oxygenation (ECMO) (one study^11^) were rarely reported. Low tidal volume ventilation was mentioned in two studies.^11,15^ Adjunctive therapies included antibiotics and steroids (see Supplementary file).

### ICU stay and patient outcomes

Mortality rates varied widely, ranging from 22% to 77%. A multicohort study reported 22% mortality in WHO-SRD patients,^16^ while South African ICU patients had a 30% mortality rate.^17^ Ugandan AHRF patients faced the highest mortality (77%, rising to 85% at 90 days).^14^

The mortality of patients with COVID-19 ARDS was 51.9% in Uganda^15^ and 69.2% in South Africa.^11^ In the cohort of patients with H1N1-related ARDS in South Africa, mortality was 68.4%,^10^ while other SSA studies reported ARDS mortality between 40% and 50%.^5,13,15^

Two studies reported the predictive risk of ARDS on mortality rather than providing prevalence or outcome data. A meta-analysis from Ethiopia reported a significantly increased risk of death (odds ratio [OR] = 21.05).^20^ In Guinea,^21^ a study found ARDS patients had an OR of 6.33 for mortality.

ICU stays for ARDS patients were prolonged, averaging 8–15 days, reflecting the severity of illness and resource constraints in SSA. Table 3 summarises ARDS outcomes.

**Table 3.** Mortality and length of stay for those admitted to ICU with ARDS.

**Study**	**ICU/hospital mortality for ARDS**	**ICU/hospital** L**OS for ARDS**
**Non-COVID-19 studies**		
**Koegelenberg *et al.* (2010)**	68.4%	15.5 days (SD = 11.2)
**Riviello *et al.* (2015)**	50%	19 days (IQR = 13–37)
**Osei-Ampofo *et al.* (2018)**	100% (2 patients)	4 and 11 days
**Kwizera *et al.* (2020)**	77%	Not reported
**Pisani *et al.* (2020)**	Not reported	Not reported
**Freercks *et al.* (2022)**	30%	Not reported
**Kwizera *et al.* (2022)**	42%	8.1 days (SD = 7.5)
**Chang *et al.* (2023)**	22%	Not reported
**COVID-19 studies**		
**Tolossa *et al.* (2022)**	Not reported	<11 days (47%) >11 days (52%)
**Arnold-Day *et al.* (2022)**	69.2%	19.5 days (IQR = 9–36)
**Kwizera *et al.* (2023)**	51.9%	6 days (IQR = 3–9)

Hospital value is used when both are reported. ICU: intensive care unit; ARDS: acute respiratory distress syndrome; LOS: length of stay; SD: standard deviation; IQR: interquartile range.

### Resource limitations

Several studies highlight significant challenges and limitations in critical care resources. The unavailability of essential supplies, such as ventilators, ICU beds, and advanced diagnostic techniques, was a recurrent theme across multiple settings. Some studies emphasised restricted access to ICU beds, with admissions limited to the most critically ill patients, especially during the COVID-19 pandemic.^5,11^ Others highlighted the scarcity of ventilators and human resources, and in certain cases, the consequent need for involvement of family members in patient care.^14,19^ Several studies also noted delays in ICU admission, with Tolossa *et al.* reporting that 90.6% of patients experienced >24 h delays.^12^ Moreover, ongoing challenges with electricity and oxygen supply interruptions were also noted (see Supplementary file).^15^

## Discussion

### Summary of evidence

This systematic review examined the epidemiology, management, and outcomes of ARDS in SSA from 2000 to 2024. Thirteen studies from 11 countries were identified, revealing highly variable prevalence and mortality estimates. Infectious causes predominated, though trauma and postoperative complications were also common. Challenges in diagnosis and resource limitations significantly impacted ARDS recognition and management.

### Diagnostic variability and prevalence of ARDS

The prevalence of ARDS varied widely across studies, ranging from 2.4% to 100%, largely due to differences in study design and patient populations, diagnostic criteria, and healthcare settings.

Globally, ARDS accounts for approximately 10% of admissions to the ICU and 23% of those mechanically ventilated.^2^ However, comparing ARDS prevalence between resource-limited and resource-rich settings is challenging due to methodological differences across studies. Although infection and trauma are more frequent in low-income countries, ARDS may also be more commonly diagnosed in high-income countries (HICs), where IMV is more accessible and can itself contribute to ARDS through ventilator-induced lung injury (VILI).^4^

Notably, in Uganda, while 70% of patients met the Kigali-modified ARDS criteria, only 17% received a clinical diagnosis of ARDS, highlighting difficulties in recognition of this syndrome and the necessity of proper training.^13^ Underdiagnosis of ARDS is a recognised issue in both resource-limited and resource-rich countries.^3^ The Berlin definition, requiring ABG analysis and CXR, may not be feasible in low-resource settings. The Kigali modification addresses these limitations and has been widely adopted in SSA.^7^ This modified definition defines ARDS based on the presence of bilateral opacities seen on LUS or CXR and hypoxia. SpO_2_ is used instead of ABGs, and the need for PEEP is removed. However, its use complicates inter-study comparisons and poses additional limitations.^7^ For instance, the SpO_2_/FiO_2_ is not a good index of severity of gas exchange when SpO_2_ is higher than 97% because of the shape of the oxyhaemoglobin dissociation curve. Moreover, SpO_2_ measurement could be inaccurate in individuals with dark skin.^7^

### Demographics

Patients with ARDS in SSA were younger (median 37–54 years) than typically reported in high-income settings, where the median age often exceeds 60 years.^3^ Most studies found a male predominance, although a South African H1N1 cohort reported nearly 80% female patients, many of whom were pregnant.^10^ Pregnancy, obesity, and DM are recognised as potential risk factors for severe H1N1 influenza.^10^

Co-morbidities associated with ARDS, such as DM, HTN, and HIV, are prevalent in SSA,^11,12^ and may exacerbate the severity and outcome of respiratory illness. In Uganda, nearly one-third of ARDS patients were HIV-positive, highlighting the importance of integrated management strategies.^14^

The most common predisposing events included infection, trauma, postoperative complications, and neurological disease. These patterns differ somewhat from HICs, where infection predominates but trauma contributes less.^5,22^

### Management and supportive therapies

Oxygen support was inconsistently reported, and access to IMV was limited. Research indicates that IMV in low-income settings may be associated with higher mortality rates compared to HICs.^23^ This disparity may stem from insufficient training, resource constraints, and the lack of essential safety equipment. For example, end-tidal carbon dioxide monitoring, which is critical for patient safety during IMV, is often unavailable due to its prohibitive cost.^23^

Adjunctive therapies such as corticosteroids, neuromuscular blockade, and proning were rarely used.^11,15,19^ The limited use of such adjunctive strategies may reflect both a lack of resources and limited training in their application, underscoring the need for further education and infrastructure improvements in critical care. Riviello *et al.* argued that some interventions considered safe in HICs might not be so in low-resource settings.^4^ For instance, proning is theoretically possible given that it requires no particular technology; however, the ability to perform it safely with the few staff available may be a barrier.^4^ VV-ECMO, an established supportive therapy used in patients with severe ARDS in HICs,^2^ was reported exclusively in South Africa, where it was used in six patients with COVID-19-related ARDS.^11^

### Patient outcomes and mortality

Reported mortality from ARDS in SSA ranged from 22% to 77%. These outcomes are comparable to global estimates but are likely exacerbated by delayed hospital admission, shortages of ICU beds, and limited availability of advanced therapies.^3^ It is important to note that four studies focus on COVID-19-related ARDS. The COVID-19 pandemic placed unprecedented strain on healthcare systems globally, including well-resourced systems in HICs. This likely contributed to poorer outcomes in these studies.

### Implications for practice and policy

This review underscores the urgent need to expand and improve ICU services in SSA. Standardising ARDS diagnostic criteria, including validation of the Kigali definition, is essential for accurate diagnosis and treatment. Capacity-building initiatives should focus on training healthcare providers in IMV, low tidal volume strategies, and adjunctive therapies.

Emerging technologies, such as artificial intelligence (AI)-driven decision-support systems and portable diagnostic devices, offer opportunities to enhance critical care in low-resource settings. Future research should prioritise regional epidemiology, clinical interventions, and translational studies to address ARDS management challenges in SSA effectively.^24^

### Limitations

This review has several limitations. First, it is based on a small number of studies conducted over two decades, many of which had small sample size and single-centre designs, limiting the generalisability of their findings to broader SSA populations. Additionally, several studies were retrospective, relying on incomplete or inconsistent medical records, which introduces bias and affects data accuracy. Heterogeneity in these data could be exaggerated by gaps in research coverage, particularly from countries with very limited healthcare resources.

Outcome reporting was also inconsistent, particularly regarding mortality and length of stay, complicating comparisons between studies. Additionally, most studies were conducted in tertiary care hospitals or ICUs, potentially skewing results by overrepresenting the most severe cases while underrepresenting community-level or milder cases, introducing selection bias.

Finally, the lack of detailed reporting on specific interventions, such as IMV, adjunctive treatment, and NIV practice, makes it difficult to assess the feasibility of applying global ARDS management guidelines in SSA.

## Conclusions

This systematic review underscores the significant burden of ARDS in SSA, highlighting the urgent need to address data gaps and improve clinical management strategies. Strengthening healthcare infrastructure, including access to diagnostic tools and critical care resources, should be a priority. Additionally, implementing standardised diagnostic criteria tailored to low-resource settings will enhance the recognition and treatment of ARDS.

## Supplemental Material

sj-docx-1-shr-10.1177_20542704251390024 - Supplemental material for Epidemiology, management and outcome of acute respiratory distress syndrome in Sub-Saharan Africa: a systematic review Supplemental material, sj-docx-1-shr-10.1177_20542704251390024 for Epidemiology, management and outcome of acute respiratory distress syndrome in Sub-Saharan Africa: a systematic review by Valentina Camarda and Robert F. Miller in JRSM Open

sj-docx-2-shr-10.1177_20542704251390024 - Supplemental material for Epidemiology, management and outcome of acute respiratory distress syndrome in Sub-Saharan Africa: a systematic review Supplemental material, sj-docx-2-shr-10.1177_20542704251390024 for Epidemiology, management and outcome of acute respiratory distress syndrome in Sub-Saharan Africa: a systematic review by Valentina Camarda and Robert F. Miller in JRSM Open
