# Supplementary material for: Epidemiology, management and outcome of acute respiratory distress syndrome in Sub-Saharan Africa: a systematic review
Source: JRSM Open. 2025 Nov 6;16(9):20542704251390024. doi: 10.1177/20542704251390024 (PMC12592648; doi:10.1177/20542704251390024)
Supplement: sj-docx-1-shr-10.1177_20542704251390024 - Supplemental material for Epidemiology, management and outcome of acute respiratory distress syndrome in Sub-Saharan Africa: a systematic review [file sj-docx-1-shr-10.1177_20542704251390024.docx]

## Epidemiology, management and outcome of Acute Respiratory Distress Syndrome in Sub-Saharan Africa: A systematic review.

## Supplemental material

### Research Strategy

**PUBMED**

(Acute respiratory distress syndrome [MeSH:NoExp] OR Acute Lung Injury[MeSH:NoExp] OR "Respiratory distress syndrome" OR "Adult respiratory distress syndrome" OR Respiratory Distress Syndrome[MeSH:NoExp] OR ARDS[MeSH:NoExp] ) AND ("Sub-Saharan Africa" OR Angola OR Benin OR Botswana OR Burkina Faso OR Burundi OR Cabo Verde OR Cameroon OR Central African Republic OR Chad OR Comoros OR Congo OR Democratic Republic of the Congo OR Djibouti OR Equatorial Guinea OR Eritrea OR Eswatini OR Ethiopia OR Gabon OR Gambia OR Ghana OR Guinea OR Guinea-Bissau OR Ivory Coast OR Kenya OR Lesotho OR Liberia OR Madagascar OR Malawi OR Mali OR Mauritania OR Mauritius OR Mozambique OR Namibia OR Niger OR Nigeria OR Rwanda OR Sao Tome and Principe OR Senegal OR Seychelles OR Sierra Leone OR Somalia OR South Africa OR South Sudan OR Sudan OR Tanzania OR Togo OR Uganda OR Zambia OR Zimbabwe) AND (epidemiology [MeSH:NoExp] OR incidence [MeSH:NoExp] OR prevalence OR management [MeSH:NoExp] OR ventilation OR strategy OR guidelines OR therapies [MeSH:NoExp] OR outcomes ) AND ((2000:3000/12/12[pdat]) AND (english[Filter]))

**COCHRANE**

("Respiratory Distress Syndrome" OR "Acute respiratory distress syndrome" OR "Acute Lung Injury" OR "Respiratory distress syndrome" OR "Adult respiratory distress syndrome" OR ARDS):ti,ab,kw AND ("Sub-Saharan Africa" OR Angola OR Benin OR Botswana OR "Burkina Faso" OR Burundi OR "Cabo Verde" OR Cameroon OR "Central African Republic" OR Chad OR Comoros OR Congo OR "Democratic Republic of the Congo" OR Djibouti OR "Equatorial Guinea" OR Eritrea OR Eswatini OR Ethiopia OR Gabon OR Gambia OR Ghana OR Guinea OR "Guinea-Bissau" OR "Ivory Coast" OR Kenya OR Lesotho OR Liberia OR Madagascar OR Malawi OR Mali OR Mauritania OR Mauritius OR Mozambique OR Namibia OR Niger OR Nigeria OR Rwanda OR "Sao Tome and Principe" OR Senegal OR Seychelles OR "Sierra Leone" OR Somalia OR "South Africa" OR "South Sudan" OR Sudan OR Tanzania OR Togo OR Uganda OR Zambia OR Zimbabwe):ti,ab,kw AND ("Epidemiology" OR incidence OR prevalence OR management OR ventilation OR strategy OR guidelines OR therapies OR outcomes):ti,ab,kw

**EMBASE**

1 ("Acute respiratory distress syndrome" or "Acute Lung Injury" or "Respiratory distress syndrome" or "Adult respiratory distress syndrome" or "Respiratory Distress Syndrome" or ARDS).mp. [mp=title, abstract, heading word, drug trade name, original title, device manufacturer, drug manufacturer, device trade name, keyword heading word, floating subheading word, candidate term word]

2 limit 1 to (english language and yr="2000")

3 ((("Sub-Saharan Africa" or Angola or Benin or Botswana or Burkina Faso or Burundi or Cabo Verde or Cameroon or Central African Republic or Chad or Comoros or Congo or Democratic Republic of the Congo or Djibouti or Equatorial Guinea or Eritrea or Eswatini or Ethiopia or Gabon or Gambia or Ghana or Guinea or Guinea-Bissau or Ivory Coast or Kenya or Lesotho or Liberia or Madagascar or Malawi or Mali or Mauritania or Mauritius or Mozambique or Namibia or Niger or Nigeria or Rwanda or Sao Tome) and Principe) or Senegal or Seychelles or Sierra Leone or Somalia or South Africa or South Sudan or Sudan or Tanzania or Togo or Uganda or Zambia or Zimbabwe).mp. [mp=title, abstract, heading word, drug trade name, original title, device manufacturer, drug manufacturer, device trade name, keyword heading word, floating subheading word, candidate term word]

4 (epidemiology or incidence OR prevalence or management or ventilation or strategy or guidelines or therapies or outcomes).mp. [mp=title, abstract, heading word, drug trade name, original title, device manufacturer, drug manufacturer, device trade name, keyword heading word, floating subheading word, candidate term word]

5 1 and 3 and 4

### Tables and figures

**Figure S1**

Figure S1: Newcastle-Ottawa Scale for cohort studies. Maximum value =9

**Figure S2**

Figure S2: JBI Critical Appraisal Tool for Cross-sectional studies. Maximum value = 8

**Table S1**

| **Study** | **Type of oxygen support** | **Adjunctive treatments** |
| --- | --- | --- |
| Koegelenberg et al. (2010) | NIV, IMV | Corticosteroids |
| Riviello et al. (2015) | 30.9% IMV | Not reported |
| Osei-Ampofo et al. (2018) | IMV | Proning and NMBA not used |
| Kwizera et al. (2020) | 6% IMV | Not reported |
| Pisani et al. (2020) | Not reported | Not reported |
| Donamou et al. (2021) | Not reported | Corticosteroids |
| Kwizera et al. (2022) | IMV | Not reported |
| Freercks et al. (2022) | Not reported | Not reported |
| Tolossa et al. (2022) | Face mask 26.6%, intranasal oxygen 73.4% | Corticosteroids |
| Endeshaw et al. (2022) | Not reported | Not reported |
| Kwizera et al. (2023) | Standard O₂ 35.3%, HFNO 9.4%, CPAP 4.8%, NIV 14.4%, IMV 36.1% | Proning, corticosteroids |
| Chang et al. (2023) | Not reported | Not reported |
| Arnold-Day et al. (2022) | IMV | Proning, NMBA, ECMO, corticosteroids |

Table S1: type of oxygen support and adjunctive treatments. NIV: Non-Invasive Ventilation; IMV: Invasive Mechanical Ventilation; HFNO: High-Flow Nasal Oxygen; CPAP: Continuous Positive Pressure Ventilation; NMBA: Neuromuscular Blocking Agent; ECMO: Extracorporeal Membrane Oxygenator

**Table S2**

| **Study** | **Reported resources limitation** |
| --- | --- |
| Koegelenberg et al.(2010) | Unavailability of ECMO. |
| Riviello et al.(2015) | Only 30.9% of patients with ARDS were admitted to an ICU. |
| Osei-Ampofo et al.(2018) | Scarcity of ventilators. |
| Kwizera et al.(2020) | Limited availability of ventilators and human resources; family members often involved in patient care; no arterial blood gas analysis or microbiological samples available. |
| Donamou et al.(2021) | Delayed ICU admissions (during Covid-19). |
| Pisani et al.(2021) | No radiological imaging techniques available to confirm LUS findings; echocardiography unavailable. |
| Kwizera et al.(2022) | Limited ICU bed capacity, with only the sickest patients admitted to ICU (Covid-19). |
| Tolossa et al.(2022) | 90.57% of patients experienced delays of over 24 hours to obtain admission (Covid-19). |
| Arnold-Day et al.(2022) | Only patients who required IMV were admitted to the ICU; limited ICU beds (Covid-19). |
| Endeshaw et al.(2022) | Lower availability of medications and medical equipment compared to high-income countries. |
| Freercks et al.(2022) | Not reported. |
| Chang et al.(2023) | Skills limitations in diagnosing ARDS, even with the use of the Kigali criteria. |
| Kwizera et al.(2023) | Only 39% of patients were admitted to ICU or HDU. Low availability of IMV; limited human and material resources in both non-ICU and ICU wards. Continuous pulse oximetry monitoring not available for all ICU beds. Power cuts and interruptions in oxygen supply. |

Table S2: resources limitations reported in the studies. ECMO: Extracorporeal Membrane Oxygenator; IMV: Invasive Mechanical Ventilation; ICU: Intensive Care Unit; HDU: High-dependency Unit; LUS: Lung Ultrasound

**Figure S3**


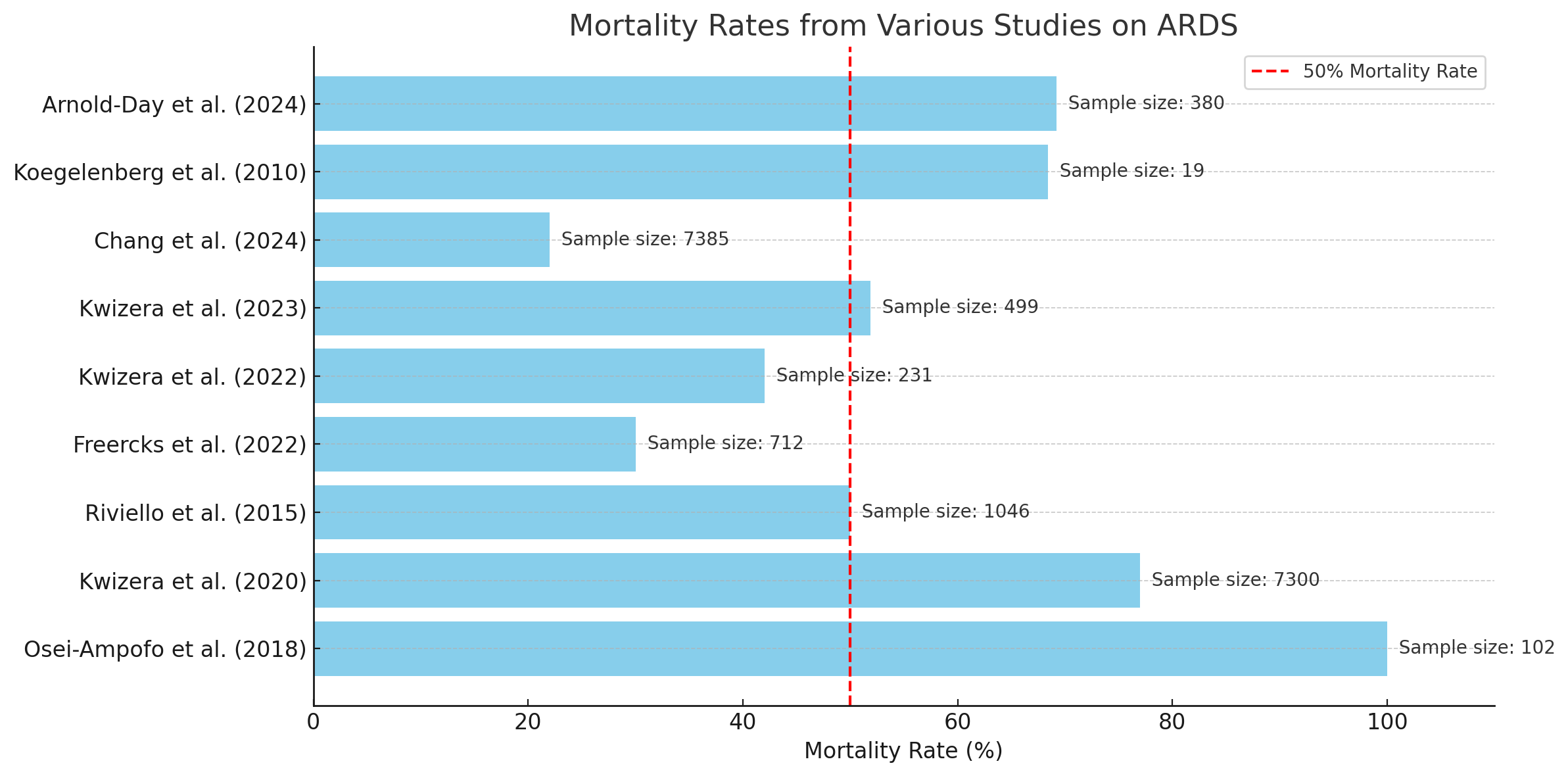
*Figure S3: Mortality reported in the different populations and sample size.*
